# Socio-demographic and lifestyle factors associated with hypertension in Nigeria: results from a country-wide survey

**DOI:** 10.1038/s41371-022-00673-1

**Published:** 2022-03-24

**Authors:** Azuka S. Adeke, Babangida S. Chori, Dinesh Neupane, James E. Sharman, Augustine N. Odili

**Affiliations:** 1 Nigeria Field Epidemiology and Laboratory Training Program, Abuja, Nigeria; 2 Alex Ekwueme Federal University Teaching Hospital, Abakaliki, Nigeria; 3 Circulatory Health Research Laboratory, College of Health Sciences, University of Abuja, Abuja, Nigeria; 4 Johns Hopkins Bloomberg School of Public Health, Johns Hopkins University, Baltimore, USA; 5 Menzies Institute for Medical Research, University of Tasmania, Hobart, Australia

**Keywords:** Hypertension, Disease prevention

## Abstract

With the rising prevalence of hypertension, especially in Africa, understanding the dynamics of socio-demographic and lifestyle factors is key in managing hypertension. To address existing gaps in evidence of these factors, this study was carried out. A cross-sectional survey using a modified WHO STEPS questionnaire was conducted among 3782 adult Nigerians selected from an urban and a rural community in one state in each of the six Nigerian regions. Among participants, 56.3% were women, 65.8% were married, 52.5% resided in rural areas, and 33.9% had tertiary education. Mean ages (SD) were 53.1 ± 13.6 years and 39.2 ± 15.0 years among hypertensive persons and their normotensive counterparts respectively. On lifestyle, 30.7% had low physical activity, 4.1% consumed tobacco currently, and 35.4% consumed alcohol currently. In comparison to unmarried status, being married (OR = 1.88, 95% CI: 1.41–2.50) or widowed (OR = 1.57, 95% CI: 1.05–2.36) was significantly associated with hypertension, compared with never married. Compared with no formal education, primary (OR = 1.44, 95% CI: 1.12–1.85), secondary (OR = 1.37, 95% CI: 1.04–1.81), and tertiary education (OR = 2.02, 95% CI: 1.57–2.60) were associated with hypertension. Low physical activity (OR = 1.23, 95% CI: 1.05–1.42), alcohol consumption, (OR = 1.18, 95% CI: 1.02–1.37), and unemployment status (OR = 1.42; 95% CI: 1.07–1.88) were also associated with hypertension. Our study indicates an association of socio-demographic and lifestyle factors with hypertension, hence, there is a need for counselling, health education and policy formulation and implementation targeting these factors to prevent and control hypertension.

## Introduction

There is a high global burden of hypertension with an estimated 1.13 billion people worldwide reported to have hypertension, with most (two-thirds) living in low- and middle-income countries (LMICs) [1]. While in 1990, high systolic blood pressure (BP) was the seventh-leading risk factor by attributable disability-adjusted life-years (DALYs), in 2019, it had become the leading risk factor [2]. The African Region of the World Health Organization (WHO) has the highest prevalence of hypertension (27%) [1]. The increase in LMICs is due mainly to a rise in hypertension risk factors in their populations [1].

Several studies have reported the increasing prevalence of hypertension in Africa [3, 4]. Nigeria, as the most populous country in Africa, is also a major contributor to the increasing burden of hypertension in the continent. Between 1995 and 2020, the estimated age-adjusted prevalence of hypertension increased from 8.5% to 32.5% [5]. A recent study also found a similar prevalence of 38% from a nationwide survey in Nigeria [6].

Current evidence shows that gaps in hypertension management were attributable to socio-demographic determinants [7–9] and lifestyle factors [10, 11]. An earlier study had suggested that demographics and lifestyle variables determined racial differences in hypertension prevalence [12]. Nigeria has a rapidly growing population with increasing urbanization and numerous ethnic groups across the country’s different regions. However, in Nigeria, the relationship between socio-demographic/lifestyle factors and hypertension is understudied.

To address the existing gaps in evidence, this study was carried out as part of the Removing the Mask on Hypertension (REMAH) study, a nationwide survey of hypertension aimed at defining the true burden of hypertension in Nigeria. Previously published articles from the REMAH study focused on the study design [13], prevalence of hypertension [6], and prevalence of dyslipidemia [14]. This study intended to assess the socio-demographic and lifestyle factors associated with hypertension in a black population. The findings from this study may be useful for planning interventions and policies to prevent and control hypertension in Nigeria and other similar settings.

## Methods

### Study design

Data were derived from a subset of the REMAH study, a cross-sectional national survey on hypertension. The details of the study design have been reported in a previous study [13]. The study population comprised adults 18 years and older who lived in selected communities. A multi-stage sampling technique was used to select participants from 12 communities across six states of Nigeria. In the first stage, one state was selected from each of the six regions of the country. In the second stage, with the aid of the administrative data of the 2015 general elections of the Independent National Electoral Commission, we selected two local government areas (LGAs) in each state, consisting of urban and rural communities. For urban communities, we selected LGAs in state capitals including Abuja Municipal Area Council for Abuja (North-central), Gombe Municipal for Gombe (North-east), Gusau for Zamfara (North-west), Onitsha for Anambra (South-east), Uyo for Akwa-Ibom (South-south), and Ibadan-North for Oyo (South-west). Gwagwalada, Akko, Bungudu, Oyi, Nsit Ubium, and Akinyele LGAs were randomly selected for sampling the rural communities in these states. In the third and fourth stages, one ward from which one polling unit was randomly selected from the rural and urban LGAs. Fieldwork was carried out between March 2017 and February 2018. Out of 4665 adults invited, 4197 consented to participate in the REMAH study; however, only 3782 of them had the required data on socio-demography and lifestyle used for this study. We complied with the Helsinki guidelines for conducting research on human participants, and the study was duly approved by the University of Abuja Teaching Hospital Human Research Ethical Committee.

### Data collection

#### Socio-demographic characteristics

Data on various socio-demographic characteristics were collected using an investigator-administered questionnaire. Marital status was grouped into married, unmarried, divorced/separated, and widowed. The area of residence was either urban or rural. Work status was categorized into government-employed, non-government-employed, self-employed, non-paid, and unemployed. Educational status was classified into no formal education, primary, secondary, and tertiary education.

#### Lifestyle measures

Trained fieldworkers administered a modified WHO STEPS questionnaire to obtain information on respondents’ socio-demographic characteristics, physical activity, tobacco use, and alcohol consumption [15]. Physical activity was assessed using the International Physical Activity Questionnaire which enquired about physical activity during work and leisure. Weekly physical activity was computed by multiplying time spent (in minutes) on a given activity in the reported week by intensity in metabolic equivalents (in MET units) corresponding to that activity: 8 METs for vigorous work or recreational activities; 4 METs for moderate work or recreational activities; and 3 METs for walking activities [16]. The total weekly activity was obtained by totaling the weekly physical activity (expressed in MET-minutes/week) of the three kinds of activities. According to the global recommendation of the WHO on physical activity, respondents had high physical activity if total weekly activity was ≥600 MET-minutes or low physical activity if <600 MET-minutes. Tobacco use was defined as current tobacco use in any form of smoking, snuffing, and ingestion. Alcohol consumption was defined as current consumption of alcohol in any form and quantity.

#### Blood pressure measurement

Blood pressure was measured by auscultation of the Korotkoff sounds at the non-dominant arm using a mercury sphygmomanometer, as previously described [6]. Participants rested in a seated position for at least five minutes, and observers obtained five consecutive BP readings at 30–60 s intervals. Systolic (phase I) and phase V diastolic BPs were measured to the nearest 2 mmHg. Standard cuffs with a 12 × 24 cm inflatable portion were used. In instances where the upper arm circumference exceeded 31 cm, larger cuffs with 15 × 35 cm bladder were used. A participant’s BP was the average of the five consecutive BP measurements. Quality control measures were applied to ensure good quality measurement of BP by training observers to avoid odd readings, consecutive identical readings and zero end-digit preference. At intervals, these parameters were examined and when significant deviations were observed, observers were retrained.

Hypertension was defined according to the 2013 guidelines of the European Society of Hypertension/European Society of Cardiology as systolic BP ≥ 140 mmHg or diastolic BP ≥ 90 mmHg or self-report treatment of hypertension using antihypertensive medications [17].

#### Data management and statistical analysis

Data were managed and analyzed using SAS software version 9.4 (SAS Institute, Cary, NC). We employed the Kolmogorov-Smirnov test to ascertain the normality of continuous variables. We used mean and standard deviation as measures of central tendencies and dispersion for normally distributed continuous variables. We further analyzed differences between the means of independent binary groups using *t*-test. Proportions were used to express all categorical variables and the differences between independent groups were analyzed using chi-square. We used logistic regression models to assess the relation of various socio-demographic and lifestyle factors with hypertension. Statistical significance was set at a significance level of *p* < 0.05.

## Results

### Characteristics of study participants

Table 1 summarizes the characteristics of the study participants. Of 3782 participants, 1654 (43.7%) were men and 2128 (56.3%) were women. Majority (2483, 65.8%) of the participants were married, 1985 (52.5%) resided in rural areas, and 1280 (33.9%) had tertiary education. Hypertensive patients were older than their normotensive counterparts. On lifestyle, 1160 (30.7%) of the participants had low physical activity, 156 (4.1%) consumed tobacco while 1340 (35.4%) consumed alcohol. Only 3.2% of the study participants consumed both alcohol and tobacco, 8.1% were physically inactive and consumed alcohol, and 1.0% were physically inactive and consumed tobacco.

**Table 1:** Characteristics of study participants.

Variable	Normotensive (%)	Hypertensive (%)	*P*-value
Socio-demographic characteristics			
Age (years)^a^	39.2 ± 15.0	53.1 ± 13.6	<0.0001
Sex			
Men	1235 (45.7)	419 (38.8)	0.0001
Women	1467 (54.3)	661 (61.2)	
Marital status			
Single	803 (29.8)	72 (6.7)	
Married	1680 (62.3)	803 (74.5)	<0.0001
Divorced/separated	32 (1.2)	17 (1.6)	
Widowed	183 (6.7)	186 (17.2)	
Area of residence			
Rural	1438 (53.2)	547 (50.6)	0.1526
Urban	1264 (46.8)	533 (49.4)	
Work status			
Government-employed	424 (15.7)	202 (18.7)	
Non-government-employed	228 (8.5)	61 (5.6)	<0.0001
Self-employed	1336 (49.5)	592 (54.8)	
Non-paid	11 (0.4)	5 (0.5)	
Unemployed	700 (25.9)	220 (20.4)	
Educational status			
No formal education	379 (14.0)	209 (19.4)	
Primary education	548 (20.3)	286 (26.6)	<0.0001
Secondary education	878 (32.5)	202 (18.7)	
Tertiary education	899 (33.2)	381 (35.3)	
Region			
North-central	518 (19.2)	101 (9.4)	
North-east	752 (27.8)	213 (19.7)	
North-west	512 (19.0)	125 (11.6)	<0.0001
South-east	236 (8.7)	196 (18.1)	
South-south	310 (11.5)	215 (19.9)	
South-west	374 (13.8)	230 (21.3)	
Lifestyle factors			
Physical activity			
High	1907 (70.6)	715 (66.2)	0.0084
Low	795 (29.4)	365 (33.8)	
Tobacco use			
Yes	120 (4.4)	36 (3.3)	
No	2582 (95.6)	1044 (96.7)	0.1218
Alcohol consumption			
Yes	928 (34.3)	412 (38.1)	
No	1774 (65.7)	668 (61.9)	0.0272
Alcohol consumption and tobacco use			
Yes	91 (3.4)	28 (2.6)	0.2173
No	2611 (96.6)	1052 (97.4)	
Alcohol consumption and physical inactivity			
Yes	188 (7.0)	117 (10.8)	<0.0001
No	2514 (93.0)	963 (89.2)	
Tobacco use and physical inactivity			
Yes	25 (0.9)	11 (1.0)	0.7896
No	2677 (99.1)	1069 (99.0)	

^a^Mean ± Standard deviation.

### Association between socio-demographic variables and hypertension

**Fig. 1:**
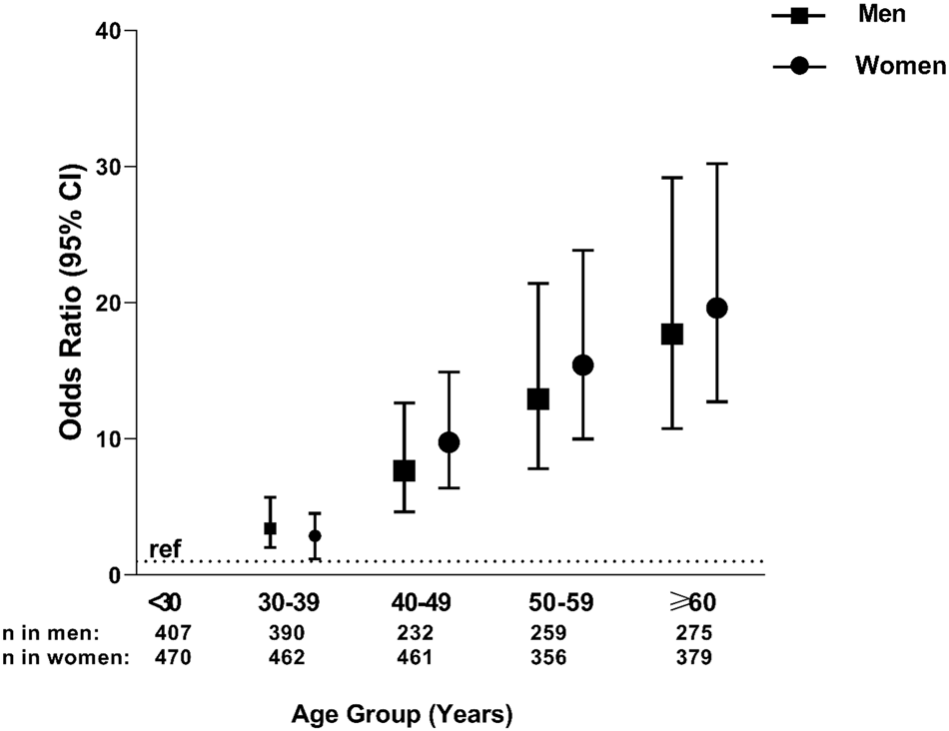
Odds ratio of hypertension by age group in men and women. The x-axis represents age group (in years) while the y-axis shows the odds ratio. The square symbol represents odds ratio for men and the circle for women.

**Fig. 2:**
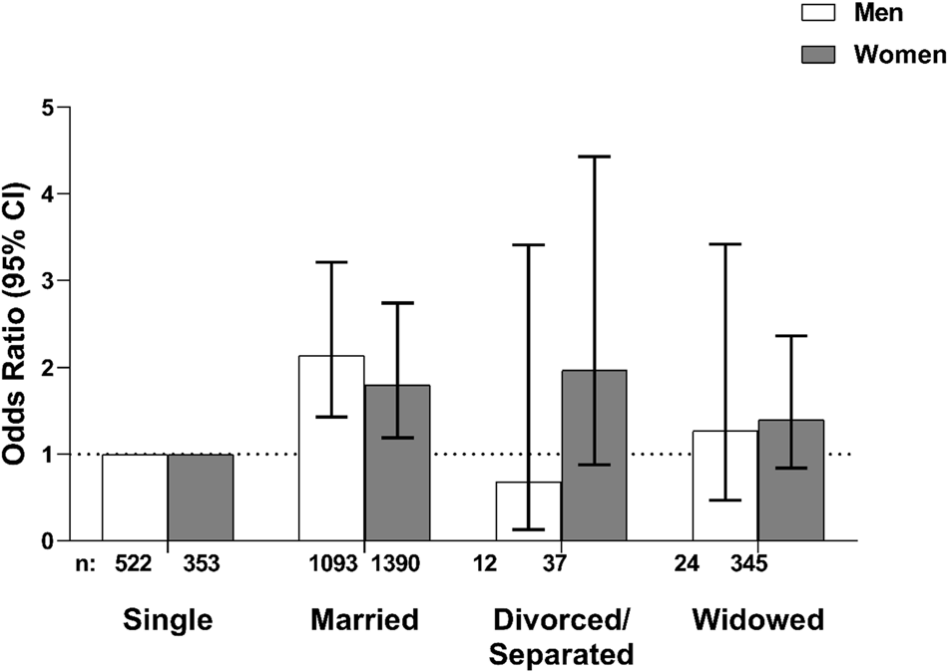
Odds ratio of hypertension by marital status in men and women (adjusted for age). The x-axis represents marital status while the y-axis shows the odds ratio. The unshaded bar represents odds ratio for men and the shaded bar for women.

Figure 1 shows the increasing positive association between different age groups and hypertension in women and men. Table 2 shows the association of other socio-demographic variables with hypertension. After adjusting for age and sex, in comparison to unmarried status, being married (OR = 1.88, 95% CI: 1.41–2.50) or widowed (OR = 1.57, 95% CI: 1.05–2.36) were positively associated with hypertension. After stratifying by sex, being married remained significantly associated with hypertension in women (OR = 1.80, 95% CI: 1.19–2.74) and men (OR = 2.14, 95% CI: 1.43–3.21) (Fig. 2). Unemployment/non-paid work was positively associated with hypertension (OR = 1.42, 95% CI: 1.07–1.88) while living in an urban area was not significantly associated with hypertension (OR = 1.11, 95% CI: 0.96–1.28). Compared with no formal education, primary (OR = 1.44, 95% CI: 1.12–1.85), secondary (OR = 1.37, 95% CI: 1.04–1.81), and tertiary education (OR = 2.02, 95% CI: 1.57–2.60) were associated with hypertension.

**Table 2:** Bivariate associations between socio-demographic variables and hypertension.

Variable	Frequency (%)	OR (95% CI)
Marital status^a^		
Single	875 (23.2)	1
Married	2483 (65.7)	1.88 (1.41–2.50)
Divorced/separated	49 (1.3)	1.61 (0.81–3.19)
Widowed	369 (9.8)	1.57 (1.05–2.36)
Area of residence		
Rural	1985 (52.5)	1
Urban	1797 (47.5)	1.11 (0.96–1.28)
Work status		
Employed	2843 (75.2)	1
Unemployed/non-paid	936 (24.8)	1.42 (1.07–1.88)
Educational status^a^		
No formal education	588 (15.6)	1
Primary education	834 (22.0)	1.44 (1.12–1.85)
Secondary education	1080 (28.6)	1.37 (1.04–1.81)
Tertiary education	1280 (33.8)	2.02 (1.57–2.60)
Region		
North-central	619 (16.4)	1
North-east	965 (25.5)	1.45 (1.12–1.89)
North-west	637 (16.8)	1.25 (0.94–1.67)
South-east	432 (11.4)	4.26 (3.20–5.67)
South-south	525 (13.9)	3.56 (2.70–4.68)
South-west	604 (16.0)	3.15 (2.41–4.13)
Physical activity		
High	2622 (69.3)	1
Low	1160 (30.7)	1.23 (1.05–1.42)
Tobacco use		
No	3626 (95.9)	1
Yes	156 (4.1)	0.74 (0.51–1.08)
Alcohol consumption		
No	2442 (64.6)	1
Yes	1340 (35.4)	1.18 (1.02–1.37)

*OR* odds ratio, *CI* confidence interval.

^a^Adjusted for age and sex.

### Association between lifestyle variables and hypertension

Table 2 shows the association between lifestyle and hypertension. Low physical activity was associated with hypertension by 23% (OR = 1.23, 95% CI: 1.05–1.42). Also, alcohol consumption was associated with hypertension (OR = 1.18, 95% CI: 1.02–1.37).

## Discussion

The key findings of our study showed that some socio-demographic and lifestyle factors were associated with hypertension. As age of participants increased, there was increasing association with hypertension. Being married, widowed, unemployed/non-paid, having higher education, low physical activity, and alcohol consumption were significantly associated with hypertension.

Over the years, there has been an increase in the burden of hypertension in Nigeria. A recent systematic review reported an increase from 8.2% in 1990 to 32.5% in 2020 [5]. A previous publication from the REMAH study found the prevalence of hypertension was 38% [6]. Findings from a meta-analysis in Africa showed an estimated prevalence of 57% in an older adult population ≥50 years which may indicate the increasing burden of hypertension with increasing age [3], just as our study noted the increasing association of hypertension with increase in age.

In our study, we found that marital status was associated with the prevalence of hypertension. Being married and widowed increased the odds of having hypertension by 88% and 57% respectively in both men and women. In contrast to previous studies in Iran [18] and Poland [19], it was observed that married men have lower BP than their unmarried counterparts. The authors suggested that married men had better sleep, less stress, better moods and have a more healthy diet compared with unmarried men [18]. The study in Iran reported that married women have higher BP than the unmarried women. It has been reported that married women get stressed from taking care of their families [20].

A recent study in Ghana also explored the association of marital status with hypertension within sub-Saharan Africa [21]. Its findings showed that marital status was an independent risk factor for hypertension in Ghana for women but not for men, after controlling for lifestyle and socio-demographic factors. Our study showed reduced but significant association between marital status and hypertension for both women and men after adjusting for age. Possible explanations for this association among married men and women may be related to the social causation hypothesis. Within the Nigerian context, marriage is seen as an achievement that may influence one’s socioeconomic conditions. With improved socio-economic status, there is a tendency towards purchasing foods away from home which are likely to be more of processed foods [22] with the increased risk of hypertension. Also, roles in the marriage could put more pressures on both women and men. In Nigeria, a married woman has to combine work with her domestic responsibilities of catering for her spouse and children [23]. A married man may have to take more responsibility to provide for the needs of his family [24]. All these may contribute to stress that can increase the risk of hypertension, thereby altering the potential emotional benefits of marriage.

Another socio-demographic factor we found to be associated with hypertension was education. Educational attainment is said to be a strong measurable indicator of socio-economic status and it is usually fixed after young adulthood [25]. Previous studies from developed countries have reported that lower education tends to increase the risk of having hypertension [26, 27]. These studies found that higher education may influence better awareness of hypertension, dietary and occupational choices. However, our study observed a higher association of tertiary education with hypertension. In the Nigerian context, attaining tertiary education may be linked with better occupational and economic opportunities and the tendencies towards urban lifestyles such as sedentary living, eating unhealthy foods, as well as engaging in more work to pay bills.

Physical inactivity is a growing concern as a risk factor for cardiovascular diseases including hypertension due to increasing urbanization and the tendency for sedentary lifestyles. We found an association of low physical activity with hypertension in our study. Recent studies continue to emphasize the beneficial effects of physical activity in the prevention and control of hypertension [28–30]. The WHO suggests that policies to increase physical activity should aim to ensure that among other measures, walking, cycling and other non-motorized forms of transport are accessible and safe for all [31]. In Nigeria, there is a plan for a national non-motorized transport policy with focus to improve access for walking and cycling as most Nigerian roads lack walkways, with pedestrians and cyclists sharing the roadway with motorized transport [32]. The current road architecture greatly discourages walking and cycling as forms of physical activity due to the dangers posed by motorized transport.

Furthermore, our study reported an association of alcohol consumption with hypertension. It has been well established in the literature that alcohol consumption increases the risk of hypertension. A recent systematic review buttressed that reducing intake of alcohol lowers BP in a dose-dependent pattern [33]. This emphasizes the importance of alcohol policies to reduce alcohol consumption. It has been reported recently that Nigeria has few alcohol-related policies with weak multi-sectoral action and funding constraint for their implementation and enforcement [34]. These policies address the need for limitation of access to alcohol, although, tax increase on alcohol and prohibition of alcohol advertisement were not addressed. With these policy gaps, there is need for more attention on alcohol control by developing a comprehensive policy to regulate its harmful use.

Our findings may be generalised to other countries of sub-Saharan Africa, as most countries within the sub-region are undergoing demographic transition with implications for health. There is an ongoing population increase with an associated increase in the aging population while still having a large young population [35]. Although there is rapid urbanization in most countries within the sub-region, physical infrastructure that encourages physical exercise is lacking in most cities. This, coupled with poor regulation of consumption of alcohol and sugar-sweetened beverages may contribute to fuel the epidemic of hypertension in the region.

One prominent strength of our study is its large sample size with participants recruited from the six regions of Nigeria. Hence, the findings of our study may be used to plan interventions or policies for the prevention and control of hypertension among similar populations. The results of this study should be interpreted within the context of the potential limitations. Our study was a cross-sectional study and hence, the findings do not infer causation in relation to socio-demographic/lifestyle factors and the prevalence of hypertension. A repeat BP measurement after at least two weeks apart would have ensured true diagnosis of hypertension according to the guideline. We, however, averaged five BP readings which may approximate closely to an individual’s usual BP. Furthermore, we deployed standardized methodology to ensure good quality of BP measurement throughout the entire period of the survey so as to appropriately identify cases of hypertension. Digital devices may be considered in future studies to improve the quality of BP measurement. Also, some of the variables were assessed through participants’ self-reporting and this might have had potentials to bias the findings of this study. Variables such as physical activity, tobacco use and alcohol consumption were prone to self-reporting bias, even though we employed trained research assistants to interview participants. In addition, tobacco use and alcohol consumption were not quantified; quantifying them may have generated dose-response association with hypertension in this study. Another key limitation in our study is the lack of data on participants’ income, an important socio-demographic variable. The lack of data on income may have limited our findings on the association of socio-economic status and hypertension as we used education as an indicator of socio-economic status in our study.

## Conclusion

In conclusion, we have reported the socio-demographic and lifestyle factors associated with the prevalence of hypertension in Africa’s most populous country. Marriage, education, low physical activity, and alcohol consumption were significantly associated with hypertension. These may be associated with more cases of hypertension presenting to health facilities, with a rising burden of the disease. Hence, there is a need for counselling, health education and policy formulation and implementation targeting these factors to prevent and control hypertension. Nurses and community health extension workers should be trained on counselling in line with the task-sharing policy. Also, the plan for a national non-motorized transport policy in Nigeria with focus to improve access for walking and cycling should be expedited by both federal and state governments. On alcohol consumption, there is need for more attention on alcohol control through development of a comprehensive policy to regulate its harmful use and improve multi-sectoral action and funding for enhanced implementation of policy.

Future research efforts include use of religious bodies to raise awareness of hypertension as well as serving as medium for counselling and health education on hypertension. The focus will be on the findings of the study which include marriage, education, physical activity, and alcohol consumption.

### Summary table

#### What is known about topic

- Socio-demographic and lifestyle factors have been reported to be associated with hypertension in some studies in high-income countries
- Most Nigerian studies focused on prevalence of hypertension at subnational levels or within small populations

#### What this study adds

- We identified a higher prevalence of hypertension among married people and those with higher educational status among adult Nigerians
- Low physical activity and alcohol consumption were also associated with hypertension among adult Nigerians

## Data Availability

The dataset used in this study is available from the corresponding author on reasonable request.
